# Molecular Landscape and Therapeutic Strategies in Cholangiocarcinoma: An Integrated Translational Approach towards Precision Medicine

**DOI:** 10.3390/ijms22115613

**Published:** 2021-05-25

**Authors:** Marco Casadio, Francesca Biancaniello, Diletta Overi, Rosanna Venere, Guido Carpino, Eugenio Gaudio, Domenico Alvaro, Vincenzo Cardinale

**Affiliations:** 1 Department of Translational and Precision Medicine, Sapienza University of Rome, Viale dell’Università 37, 00185 Rome, Italy; marco.casadio@uniroma1.it (M.C.); rosanna.venere@uniroma1.it (R.V.); domenico.alvaro@uniroma1.it (D.A.); 2 Department of Anatomical, Histological, Forensic Medicine and Orthopedics Sciences, Sapienza University of Rome, Via Borelli 50, 00161 Rome, Italy; diletta.overi@uniroma1.it (D.O.); eugenio.gaudio@uniroma1.it (E.G.); 3 Department of Movement, Human and Health Sciences, Division of Health Sciences, University of Rome “Foro Italico”, Piazza Lauro de Bosis 6, 00135 Rome, Italy; guido.carpino@uniroma1.it; 4 Medical-Surgical and Biotechnologies Sciences, Polo Pontino, Sapienza University of Rome, Corso della Repubblica 79, 04100 Latina, Italy; vincenzo.cardinale@uniroma1.it

**Keywords:** cholangiocarcinoma, precision medicine, molecular signatures, targeted therapy

## Abstract

Cholangiocarcinomas (CCAs) are heterogeneous biliary tract malignancies with dismal prognosis, mainly due to tumor aggressiveness, late diagnosis, and poor response to current therapeutic options. High-throughput technologies have been used as a fundamental tool in unveiling CCA molecular landscape, and several molecular classifications have been proposed, leading to various targeted therapy trials. In this review, we aim to analyze the critical issues concerning the status of precision medicine in CCA, discussing molecular signatures and clusters, related to both anatomical classification and different etiopathogenesis, and the latest therapeutic strategies. Furthermore, we propose an integrated approach comprising the CCA molecular mechanism, pathobiology, clinical and histological findings, and treatment perspectives for the ultimate purpose of improving the methods of patient allocations in clinical trials and the response to personalized therapies.

## 1. Introduction

Cholangiocarcinoma is a complex nosological entity, including a heterogeneous group of biliary tract malignancies, which represents the second most common primary liver cancer and accounts for 3% of all gastrointestinal neoplasms worldwide [1]. CCA incidences show wide geographical differences, reflecting the exposure to different risk factors, such as *O. viverrini* and *C. sinensis* infections in some Eastern countries. In Western countries, the incidence of CCA, taken together, is estimated to be <four cases/100,000 people/year, while it is significantly higher in Asian countries, with peaks of 85 cases/100,000 people/year observed in Northern Thailand [2]. Over the years, several classifications have been proposed. Based on the anatomical location, three types of cholangiocarcinoma can be distinguished: intrahepatic (iCCA), perihilar (pCCA) and distal (dCCA). Histologically, pCCA and dCCA are mainly mucinous adenocarcinomas, while iCCA is characterized by a marked heterogeneity, since tumors can be conventional mucinous adenocarcinomas (large duct-type iCCA), similar to p/dCCA, or could resemble transformed interlobular bile ducts (small duct-type iCCA). These histological findings may be explained considering cholangiocarcinogenesis as a process that starts from different cells of origin. Cylindric cholangiocytes, mucous cells and peribiliary glands (PBGs) within the larger bile ducts have been proposed as cancer-initiating cells of large duct-types iCCA, pCCA and dCCA; differently, cuboidal cholangiocytes in smaller bile ducts and Hepatic Stem Cells (hHpSCs) within the canals of the Hering niche may have the same role in small duct-type iCCA [3,4]. The debate about CCA cells of origin is still ongoing, and many questions concerning cholangiocarcinogenesis and CCA heterogeneity remain open. It is known that cholestasis and/or chronic inflammation constitute the main pathogenetic mechanism, and several risk factors have been identified, but their correlation to the molecular and histological patterns has yet to be completely clarified. A recent comprehensive meta-analysis revealed that, in Asian countries, cirrhosis and HBV are the conditions mostly associated with iCCA, while, for HCV and alcohol, no statistically significant geographical differences were observed [5]. Nevertheless, CCA often occurs in the absence of known risk factors, and the diagnosis is usually late because of the insidious onset and the limitations of available diagnostic tools, so that the patients are not eligible for surgical resection, which currently represents the only curative treatment. The CCA dismal prognosis, with a 5-years survival rate of 7–20% [6], is worrisome and, together with the increasing incidence reported for iCCA, explains the endeavor to understand their pathobiology, in order to develop more effective therapeutic strategies. In fact, cisplatin–gemcitabine chemotherapy is often the only possible therapeutic option, while, in the event of failure of the first-line regimen, there is still no clear indication of a second-line regimen to date [7], although promising results have been observed in randomized controlled trials (RCT), e.g., phase II ABC-06 RCT [8]. Several authors [9,10,11] have underlined the need to overcome this oncology-based approach, and clinical trials have explored the possibility of targeted therapies [12,13], revealing the potential of precision medicine in CCA [14,15], defined as tailoring the treatment to the individual characteristics of each patient. Nevertheless, high intertumoral heterogeneity and its low incidence hinders the recruitment of large cohorts of patients who could benefit from a specific therapy. Moreover, this heterogeneity is often neglected in patient allocations in clinical trials. Some compounds have been tested in basket trials [13,16], also known as bucket trials, where the cohort entails patients suffering from different types of cancers harboring the same mutation. Other therapies have been evaluated in umbrella trials [17], where patients with the same type of cancer receive different drugs, based on their mutational profile. Nevertheless, molecular characterization is not always performed before allocating a patient to a given treatment [18], and, when it is performed, it is usually based on the search of a few defined somatic exosome mutations [19]. Conversely, in other settings, mutational profiling constitutes the only variable considered without taking into account the tumor histomorphology and etiological background and sometimes considering all biliary tract cancers (BTCs) as a single entity, comprising gallbladder cancer (GBC) [20]. In our opinion, an integrated molecular and morphological analysis of CCA, together with the clinical setting in which the tumor develops, would be crucial to identify distinct categories of patients and the related most appropriate targeted therapy. In this review, we aim at highlighting the latest achievements in understanding the CCA molecular mechanism, delineating the therapeutic opportunities derived from a translational and personalized approach.

## 2. CCA Molecular Landscape

Molecular profiling studies have better delineated the genomic and transcriptomic landscape of CCA. Several studies on diverse cohorts of patients have defined different mutational profiles in iCCA and p/dCCA and have identified molecular subgroups, associated with driver gene combinations and patient prognosis [21,22].

Nakamura et al. [23] performed whole-exome and transcriptome sequencing in a large cohort of 260 Japanese patients, including 145 with iCCA, 86 with p/d CCA and 29 with GBC, who underwent surgical resection. Significantly altered genes were examined and grouped according to different criteria, among which was the tumor site of origin, providing a key molecular basis for anatomical classification crucial to subsequent clinical trials. In particular, ARID1B and ELF3 mutations and PRKACA and PRKACB fusions occurred mostly in p/dCCA, whereas genetic alterations in fibroblast growth factor receptors (FGFR1-3) and isocitrate dehydrogenase (IDH1-2), BAP1 and EPHA2 mutations were found exclusively in iCCA. Furthermore, the authors analyzed the gene interactions from two different perspectives, defining five pathway modules by Reactome FI Cytoscape Plugin—KRAS (51.9%), TGF-β–SWI/SNF-MYC (40.2%), TP53 (33.9%), epigenetic (29.3%) and RB cell cycle (11.7%)—and performing unsupervised clustering. This classification was associated with anatomical locations and driver gene combinations, i.e., BAP1, IDH1 and NRAS mutations and FGFR2 fusions in Cluster 3, comprising mostly iCCA and characterized by an increase in metabolic processes. Patients belonging to Cluster 1 (eCCA) had a significant negative enrichment of the RAS and MAPKK activation signatures, while clusters 2 and 4 comprised iCCA, eCCA and GBC and were characterized by positive the enrichment of TP53, KRAS and PKA pathway mutations. Moreover, in cluster 4 were highlighted alterations in the genes involved in the immune system, cytokine activity and antiapoptotic genes. Both clusters and mutations were associated with patients’ prognosis, with a worse clinical outcome related to TP53, KRAS and ARID1 mutations and cluster 4, but other clinicopathological features were not evaluated. A similar approach was adopted by Wardell et al. [24], who analyzed 412 samples of iCCA (136), pCCA (109), dCCA (101) and gallbladder or cystic duct cancers (GBCs/CDCs) (66); 107 by whole-exome sequencing (WES); 39 by whole-genome sequencing (WGS) and 266 samples by targeted sequencing. Samples were obtained from surgical resection specimens of patients belonging to a Japanese and an Italian cohort. Significantly mutated genes were identified by Mut-SigCV, and their prevalence in the anatomical subgroups was studied. Epigenetic genes alterations, i.e., BAP1 and IDH1, were predominantly found in iCCA, whereas p/dCCA subtypes showed an enrichment of mutated cell cycle genes. ARID1A and KRAS mutations, consistent with previous findings [23], and a deletion of MUC17 at 7q22.1. negatively affected the patients’ prognosis. Furthermore, three mutational signatures were extracted by non-negative matrix factorization and compared to COSMIC signatures [25], an international dataset that includes a great number of samples from several type of cancers: Signature 1 (5-methylcytosine deamination), Signature 2 (AID/APOBEC deaminases) and Signature 5 (related to age or nucleotide excision repair deficiency) This strategy supports potential associations between signatures found in different diseases and might help attributing meaning to a specific signature. In this cohort, signature 1 was linked to aging, while Signature 5 was related to age, nucleotide excision repair deficiency and, of note, to HBV and HCV infections. 

The role of the risk factors, such as viral hepatitis and cirrhosis, was investigated with regard to molecular characterization by Sia et al. [26], who defined two main iCCA biological classes, proliferation and inflammation, by an integrative genomic analysis of 149 samples based on the gene expression profile (transcriptomic), high-density single-nucleotide polymorphism array and mutation analyses. The proliferation class was enriched for receptor tyrosine kinase (RTK) pathways, i.e., epidermal growth factor (EGF), RAS, AKT, MET and the angiogenesis-related vascular endothelial growth factor (VEGF) and platelet-derived growth factor (PDGF). Thus, the authors presented the possibility of testing sorafenib, a multikinase inhibitor poorly effective in CCA patients [20], specifically on the proliferation class, underlining the importance of characterizing the subgroup of patients to whom a given therapy should be administered. A potential targeted therapy with JAK-STAT inhibitors was suggested for the inflammation class, in which immune response-related pathways were observed, i.e., the overexpression of cytokines belonging to the Th2 subtype and deregulation of the Th1 subtype, together with a significant enrichment of nuclear pSTAT3. Interestingly, the biological classes were also analyzed with an integrated approach, with respect to the laboratory parameters, such as ALT, bilirubin, and albumin, and anatomopathological features, i.e., TNM and microvascular, intraneural and ductal invasion. The proliferation class, though, was significantly associated with a poorer histologic differentiation and intraneural invasion and, notably, with a significantly shorter overall survival and time to recurrence. Histological features were not further analyzed in this cohort, while they were better delineated by Montal et al. [27] in p/dCCA samples only. Authors are credited with spotlighting a subgroup that is usually underrepresented in international datasets, i.e., TCGA [28], and has exhibited no significant response to targeted therapy, to date. A total of 189 p/dCCA samples underwent whole-genome expression, targeted DNA-sequencing and transcriptome-based unsupervised clustering, as well as immunohistochemistry. As a result, KRAS, TP53, ARID1A and SMAD4 were found to be the most prevalent mutations, whereas IDH1 mutations occurred only in a low percentage of the cohort. Interestingly, 25% of identified genetic alterations were potential therapeutic targets. Among them, ERBB2 mutations and amplifications, were more prevalent in the proliferation class, together with the overexpression of MYC targets, cell cycle signaling and DNA repair pathways. An enrichment of Ras/MAPK and AKT/mTOR pathways and a higher Ki67 staining were also found when compared to other classes. Notably, this class showed significant similarities to both iCCA and HCC proliferation classes and, at the histopathological level, to pancreatic ductal adenocarcinoma, given the overexpression of EpCAM and cytokeratins and a higher prevalence of papillary histology and intraductal papillary neoplasm of the bile duct (IPNB). Nevertheless, no similarities were found between previously defined iCCA inflammation class [26] and p/dCCA immune class, characterized by higher lymphocytic infiltration and increased immune checkpoint expression, that may be targeted by using immune checkpoint inhibitors. Metabolic class featured deregulated metabolism of bile acids, fatty acids and xenobiotics, to which authors ascribed the acquisition of a HNF4A-driven hepatocyte-like phenotype, with positive staining to HepPar-1. Mesenchymal class, comprising tumors with EMT, TGF-β signaling activation and a desmoplastic reaction observed in pathological analysis, showed the highest prevalence and the worst clinical outcome, as an independent prognostic factor.

A different clustering method, when compared to the ones previously cited, was adopted by Nepal et al. [29], who analyzed genomic and epigenomic features of 496 iCCA samples, using whole-exome sequencing (WES), targeted-exome sequencing (TES) and genome-wide DNA methylation profiling. Most recurrently mutated genes were used to stratify the samples into four groups: KRAS, TP53, IDH1 and undetermined (Udt) group. Related pathways, patients’ prognosis and in vitro drug sensitivity were assessed for each driver gene group. IDH1 group was characterized by enrichment of BCLAF1 and metabolic pathways genes alterations, and response to RNA synthesis inhibitors. Consistently with previous findings [30], chromatin-modifier genes, i.e., ARID1A and BAP1, were significantly altered in IDH1 mutated group. KRAS and TP53 groups showed the poorest prognosis, with worst overall survival and shortest time to recurrence. The former was enriched for multiple immune-related processes (NK cytotoxicity, JAK/STAT and cytokine signaling), SMAD4, ErbB, VEGF alterations and actin rearrangements, and it was sensitive to microtubule-targeting drugs. The latter was significantly enriched for PTEN, RB1, LATS2, MAPK, WNT signaling; topoisomerase inhibitors were effective on this group. Finally, ARID1A mutations, FGFR fusions and mTOR pathway alterations significantly occurred in the undetermined group, which showed a response to mTOR inhibitors. Authors investigated a possible correlation between the mutational profile and known CCA risk factors. A significant association was found between TP53 group and HBV infection, in line with previous studies [31] that hypothesized a role of TP53 pathway in the pathogenesis of HBV-related CCA. Other risk factors as type 2 diabetes mellitus (DM2), smoking or alcohol consumption did not have a significantly associated signature.

As far as PSC is concerned, genomic and transcriptomic studies included a low percentage of patients with this condition, although it represents a predominant risk factor for CCA in Western world. To overcome this limitation, 186 PSC-BTCs tissue specimens from 11 centers in Europe and U.S. were recently analyzed by Goeppert et al. [32], who established histological, molecular and etiological correlations within PSC-BTCs. Remarkably, samples were homogeneous in terms of histological pattern independently from anatomical location, being characterized by a large duct phenotype as previously observed [33]. Goeppert’s cohort showed homogeneity also in terms of molecular profile, with no statistically significant differences between iCCA, pCCA, dCCA and GBC. No FGFR translocations and only one IDH1 mutation were detected among 60 iCCA and this subgroup showed similar mutation frequencies to pCCA and dCCA, when compared to data from previous polyetiological studies. Furthermore, PSC-BTCs mutational profile was comparable to the one previously defined in liver fluke-related CCA [34,35], with a high frequency of mutations in TP53, KRAS, SMAD4 and ROBO1/2 common to both settings. Therefore, it has been hypothesized that this molecular profile might be associated to chronic inflammation and bile homeostasis disruption, that occur both in PSC and liver fluke infection.

**Figure 1:**
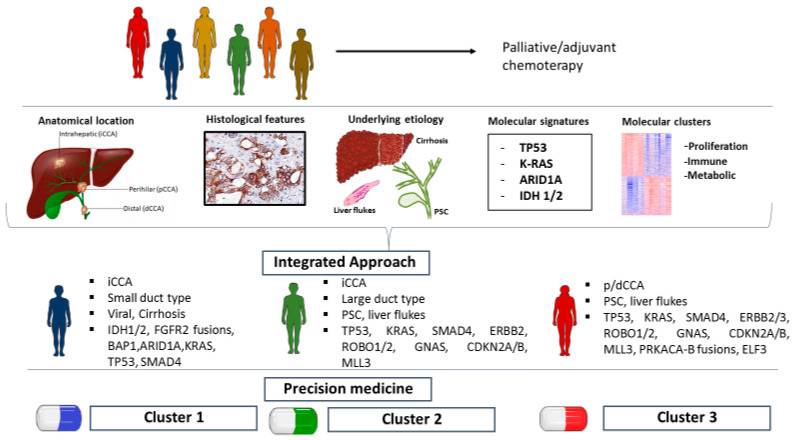
Integrated approach for CCA patient stratifications and clinical perspectives, based on the anatomical location, histological and clinical features and molecular signatures and clusters.

The hypothesis of chronic inflammation driving specific molecular alterations has been supported by Jusakul et al. [36], who carried out integrated genomic, epigenomic and transcriptomic analysis of 489 samples, both iCCA and p/dCCA, from 10 different countries, including both fluke-positive and fluke-negative patients. As a result of integrating WGS (71 cases), exome sequencing (200 cases), high-depth targeted sequencing (188 cases), SNP array copy-number profiling (175 cases), array-based DNA methylation profiling (138 cases), and array-based expression profiling (118 cases), the cohort was stratified in four clusters by using iClusterPlus. Clusters 1 and 2 were associated to fluke infection and were both significantly enriched for TP53 mutations and ERBB2 amplifications, and linked to a worse clinical outcome, when compared to Cluster 3 and 4. While Cluster 1 showed hypermethylation of promoter CpG islands, enrichment of ARID1A, BRCA1/2 and H3K27me3 mutations, cluster 2 main genetic alterations comprised upregulated CTNNB1, WNT5B and AKT1 expression and downregulation of genes involving EIF translation initiation factors. Mostly fluke-negative, Cluster 3 and 4 showed upregulation of immune checkpoint genes (PD-1, PD-L2 and BTLA), and BAP1, IDH1/2 mutations and FGFR alterations, respectively. Interestingly, Cluster 1 and 4 showed different hypermethylation patterns, probably reflecting two carcinogenesis models: the former driven by epigenetic events due to chronic inflammation, the latter based on genetic events that can drive epigenetic alterations. Furthermore, authors pointed out that anatomical site did not affect molecular subtypes nor patients’ prognosis in their cohort, since Clusters 1 and 2 comprised intrahepatic and extrahepatic tumors, while intrahepatic tumors were included in all 4 clusters. This observation, consistent with other studies [32], suggested that tumors from different anatomical site may have common molecular features, while sharing the same anatomical location does not imply molecular uniformity. Moreover, authors underlined the difficulty of merging data from multiple heterogenous cohorts and different sequencing platforms, that hampers also inter-study comparison. From our review, a marked heterogeneity in sequencing, clustering and data processing methods emerges, which needs to be added to the intrinsic cohorts’ diversity in terms of risk factors and clinicopathological features (Figure 1). These observations depict a complex landscape, that appears challenging not only to interpret but also to reproduce in experimental models. Few but well performed multicenter prospective longitudinal studies on a global scale and RCTs should be the framework to integrate diverse omics data to find a coherently matching geno-pheno-enviro-type relationship or association in CCA. 

## 3. Targeted Therapy: State-of-the-Art and Future Perspectives

CCA is a group of malignancies characterized by dismal prognosis and very high intertumoral variability. The previously described genetic and epigenetic alterations, the underlying etiology and the complex tumor microenvironment determine this variability, presumably together with different cells of origin [6].

It is therefore crucial to consider all these variables to identify the prognosis and treatment response and propose the most suitable targeted molecular agent. Unfortunately, several targeted therapies tested so far have shown only little or no overall survival benefits [37], but questions have been raised about patient allocations in appropriate trials. In many of these, it has not always been possible to take into account all these variables, and also, for this reason, the results may have been unsatisfactory [19]. In fact, the presence of an associated pathology, such as PSC, is able to modify the molecular landscape of the tumor, regardless of the anatomical location, often considered more relevant in trial patient allocations [32]. Moreover, histological and molecular stratification is not always performed, and, in some trials, CCA are considered as a single group, not even referring to the anatomical classification [20]. As previously pointed out [26], some of these compounds already tested in unstratified cohorts may have better results in molecularly and histologically defined groups.

Hereinafter, we explore the main pathways that have been targeted in clinical trials, and we analyze the criteria of patient enrollment. Furthermore, we summarize the possibility of targeted therapies, both already studied and yet to be established, with respect to molecular clusters and driver gene mutations identified in the previous sections (Table 1).

### 3.1. Metabolic Regulators 

As described above [23,29], IDH1 and IDH2 mutations are frequent in iCCA (13–25%). The inhibitors of IDH1 (AG120) and IDH2 (AG221), ivosidenib and enasidinib (NCT02273739), respectively, are currently under investigation in several trials. Abou-Alfa and colleagues, in the ClarIDHy phase III trial with 185 patients with IDH1-mutated iCCA, reported a significant risk reduction of disease progression or death with ivosidenib, with a median progression-free survival improvement and increased OS to 10.8 months, with a good safety profile [12]. D-2-dihydroxyglutarate (2-HG), an oncometabolite that accumulates in IDH1/2-mutated cells [49], was dosed before and during treatment, resulting in a drop of its concentration to almost healthy individual values after two cycles of ivosidenib. The excess of 2-HG results, indeed, in enhanced proliferation, but it also sensitizes cancer cells to DNA damage, increased by several drugs, i.e., PARP inhibitors. On this basis, the PARP inhibitor olaparib is being studied in two distinct phase II basket trials in patients with recurrent/progressive IDH1/2-mutant CCA and other solid tumors, alone (NCT03212274) and in combination with the ATR inhibitor ceralasertib (NCT03878095). Given the PARP inhibitors’ role in DNA instability, already well-established in ovarian cancer [50], niraparib has been administered in patients suffering from CCA and other malignancies in an ongoing phase II clinical trial (NCT03207347), which will evaluate the response to this treatment also in a cohort of patients carrying DNA damage response alterations, i.e., IDH1/2, BAP1, ARID1A, ATR, PTEN and other genes already well-known to play an important role in CCA. Moreover, IDH1/2 mutations have also been investigated when not directly targeted, since they induce metabolic stress in cancer cells, because of the inhibition of the tricarboxylic acid cycle (TCA) and electron transport chain (ETC) [51]. This stress can be amplified by a further ETC inhibition due to metformin, which, in a phase I/II clinical trial, has been administered together with chloroquine in order to decrease the microenvironment acidification [52]. According to the study protocol, the secondary aims would be to investigate whether 2-HG levels in biological fluids can predict both the mutational status and response to treatment, which might simplify patient allocations and follow-ups.

### 3.2. Tyrosine Kinase Receptors

As previously mentioned, almost 15–28% of iCCA exhibit gene alterations in FGFR2, demonstrating its great potential in the role as a molecular target in this setting of patients. FGFR2 inhibitors, such as pemigatinib, derazatinib (NCT03230318) and infigratinib (BGJ394), have shown promising results. In particular, pemigatinib, in an umbrella multicenter, open-label, phase II clinical trial (FIGHT-202), was administered in three groups of patients with cholangiocarcinoma, who had FGFR2 fusions or rearrangements, other FGFR2 alterations and a FGFR2 wild type. In this study, the patients belonging to the first group showed an overall response rate (ORR) of 35.5%, with a median duration of response (mDOR) of 7.5 months [53]. On the basis of these positive results, pemigatinib has been recently approved by the Food and Drug Administration (FDA) and European Medicines Agency (EMA) for previously treated, unresectable locally advanced or metastatic cholangiocarcinoma, with a FGFR2 fusion or other rearrangement. Pemigatinib is also under evaluation as a first-line treatment vs. gemcitabine plus cisplatin in patients with advanced CCA, with FGFR2 rearrangements in a phase III study (FIGHT-302) (NCT03656536) [54]. Infigratinib (BGJ398) is currently being evaluated in Phase III clinical trial as a first-line treatment in patients with advanced cholangiocarcinoma with FGFR2 gene fusions/translocations (NCT03773302). However, it has been observed that further mutations in FGFR may confer a resistance to the aforementioned FGFR inhibitors [55]. Interestingly, many point mutations in FGFR2 have been detected in cell-free DNA in CCA patients who showed an association with resistance to the pan-FGFR inhibitor infigratinib, highlighting the importance of developing strategies to overcome chemoresistance. For this purpose, futibatinib has shown promising results in patients who progressed after treatment with FGFR2 inhibitors. A further partial response has recently been observed in a patient treated with futibatinib who showed disease progression after developing an acquired L618F FGFR2 kinase domain mutation following a treatment with an oral FGFR-1/2/3 inhibitor (Debio 1347) [56].

Due to this promising evidence [57], its safety and activity in previously treated patients, Futibatinib is currently being evaluated as a first-line treatment in patients with advanced CCA in a phase III clinical trial (FOENIX-CCA3 and NCT04093362). Although the results of these studies are not conclusive yet, this finding could play an important role in the therapeutic and prognostic algorithm of this category of patients, who may be selected in the near future by noninvasive assessment [58].

The overexpression of vascular endothelial growth factor receptor (VEGFR) has been demonstrated in 31.4–75.6% of iCCA and p/dCCA, and the presence of EGFR and VEGFR family members in BTC is linked to a negative prognostic role [59]. The inhibition of these pathways has shown antitumor activity in vitro and in vivo in preclinical studies [38,60].

On these bases, the efficacy of targeting VEGF with bevacizumab has been studied in a multicenter phase II trial in combination with gemcitabine and capecitabine [18]. The results showed that it did not improve the survival in an unselected group of BTC patients with advanced or metastatic disease, so that its future use in CCA may not disregard the identification of reliable biomarkers. The multikinase inhibitor sorafenib, also targeting VEGFR2 and 3, has been evaluated in advanced CCA in different clinical trials, with no encouraging results [39]. A phase II clinical trial showed increased toxicity, with no benefits to the PFS with the addition of sorafenib to GEM-CIS in biliary tract cancer [20]. However, the authors pointed out that the trial included biliary adenocarcinomas of all subtypes, regardless of the differences in the pathology and molecular landscape, suggesting that a lack of patient stratification may be associated with suboptimal outcomes [20].

Regorafenib has shown promising result in patients with advanced BTC, with progression on the standard first-line therapy with a PFS of 15.6 weeks and OS of 31.8 weeks in this poor prognosis population, and confirmatory phase III studies are warranted [41].

### 3.3. Epidermal Growth Factor Receptor

To date, EGFR inhibitors did not show encouraging results when administered in CCA clinical trials. Several trials have explored the efficacy of combination regimens of monoclonal antibodies with chemotherapy, with mixed results.

A phase II clinical trial evaluated the efficacy of cetuximab in combination with GEMOX vs. GEMOX alone in patients with advanced BTC [19]. The patients were stratified by KRAS mutation; EGFR expression; NRAS and BRAF mutations and the tumor anatomical location (iCCA, eCCA and GBC). The study failed to demonstrate a significantly therapeutic efficacy of the combination therapy with cetuximab. A possible limitation of the study was the lack of a complete signature analysis of tumors, considering that gene alterations such as FGFR2 translocation and ROS1 fusion might lead to anti-EGFR therapy resistance [19].

The combination of panitumumab, a monoclonal anti-EGFR1 antibody, with gemcitabine and irinotecan showed good tolerability and encouraging results, with a median PFS of 9.7 months and median OS of 12.9 months [42]. These encouraging results were not confirmed in another phase II study in which panitumumab, in combination with GEMOX, failed to show an improvement of the PFS and OS in chemotherapy-naive patients with advanced BTC tested by IHC, PCR and Sanger sequencing for KRAS, BRAF and PI3KCA to identity a subgroup of patients who would not respond to anti-EGFR treatment. Nevertheless, the cohort was not specifically tested for enrichment in EGFR alterations [43].

Other studies in the past years did not show any clear benefits in terms of the OS from a combination of chemotherapy regimens with lapatinib [48] or erlotinib [44,45], while a phase II/III is currently ongoing for testing the efficacy of varlitinib, a competitive inhibitor of the tyrosine kinases EGRF and HER2–4, plus capecitabine in advanced BTC (NCT03093870).

Several solid malignancies, such as breast and gastroesophageal cancers [61], have known associations with ERBB2 gene aberrations, and anti-HER2 is currently used in clinical practice. ERRB2 aberrations are observed frequently in p/dCCA, but anti-HER2 is not yet recommended in this setting of patients [6]. For this purpose, the results of the phase II clinical trials evaluating the efficacy of trastuzumab in combination with capecitabine in patients with HER2-positive CCA are awaited with growing interest (NCT03613168).

### 3.4. PI3k/AKT/mTOR Pathway

The PI3k/AKT/mTOR pathway is implicated in the metabolism, cell cycle and survival. Alterations of this pathway have been observed in several solid tumors, including iCCA and p/dCCA [26,27,29]. Numerous preclinical in vivo and in vitro studies have shown compelling results on the efficacy of PI3K/AKT/mTOR inhibitors in BTC [46]. However, the clinical studies so far conducted have not confirmed these results. In the phase II study RADIChol [37], the mTOR inhibitor everolimus was administered as the first-line monotherapy to 27 advanced BTC patients. This study showed a median OS of 9.5 months in patients treated with everolimus, lower than the OS reached with the standard of care (11.7 months). In another phase II study (ITMO), everolimus was administered to 39 patients with progressing disease after CHT, achieving a disease control rate of 44.5% [40]. The main difference between these two studies relied on the evaluation of the activation status of the targeted pathway, which was explored only in RADIChol: several pathway components were analyzed by IHC, whereas KRAS and PIK3CA were the only two genes investigated by PCR and Sanger sequencing. No statistically significant associations were found between the clinical outcome and IHC markers, while the low prevalence of KRAS and PIKCA alterations hindered any association. These results suggested the absence, to our knowledge, of reliable biomarkers predicting the sensitivity to PI3K/mTOR inhibitors and the need of further characterizations of this pathway regulation. Another strategy to target this pathway came from the AKT inhibitor MK2206, currently under study in a phase II clinical trial (NCT01425879), which also aimed at determining the correlation between genetic alterations of the pathway and the clinical response to treatment.

### 3.5. Proteasome Inhibitors

Bortezomib is a proteasome inhibitor already approved for multiple myeloma and other hematological diseases. Despite that a phase II trial did not observe a confirmed response in unstratified patients [62], leading to early discontinuation, bortezomib effectiveness in CCA is still under assessment in a phase III trial (NCT03345303), specifically including patients with iCCA and PTEN mutation/deletion. This enrollment strategy is based on preclinical observation of proteasome dependency in CCA with PTEN loss-of-function, resulting in a promising inhibition of cell proliferation in vitro and in vivo after bortezomib administration.

### 3.6. Immunotherapy

CCA is characterized by the notable presence of desmoplastic stroma that is implicated in the reduced efficacy of the immune response and in chemoresistance [63,64]. Recently, a classification based on the tumor microenvironment for iCCA has been proposed, according to which, it is possible to distinguish four variants: immunedesert, immunogenic, myeloid and mesenchymal [65]. The immunogenic subtype is characterized by an inflammatory microenvironment and increased expression of MHC 1 and 2, as well as PDL-1; clinically, it is instead associated with a more benign prognosis. Currently, the checkpoint inhibitors have been tested in the phase Ib basket trial KEYNOTE 028 [16] including patients with CCA. This subset of patients treated with pembrolizumab (PDL-1 inhibitor) showed a median progression-free survival of 1.8 months. Unfortunately, these results have not been confirmed preliminarily in the KEYNOTE 158 trial. Nevertheless, in this trial, the checkpoint inhibitors showed promising results in patients with microsatellite instability or DNA mismatch repair [66]. Only a small percentage of patients show a positive response to treatment with immune-modulating therapy, and this highlights the need to develop predictive biomarkers. It seems that microsatellite instability (MSI) is associated with a better clinical response, and in this subset of patients, immune therapy could be a valid therapeutic alternative [67]. Given that the presence of MSI is observed in an extremely low percentage of CCA, urgent attention should be paid to the need to standardize and include MSI-high patients in future CCA therapy trials [68]. Two phase III studies are currently evaluating the efficacy of the PDL-1 inhibitors pembrolizumab (NCT04003636) and durvalumab (NCT03875235), in combination with gemcitabine and cisplatin, as the first-line therapy in patients with advanced biliary tract cancer, while bintrafusp alfa is under examination in a phase II/III clinical trial (NCT04066491).

### 3.7. Future Perspectives

Currently, there is no recommendation in favor of performing molecular profiling or specific gene alterations analyses as routine in CCA patients management [69]. A retrospective single center analysis by molecular profiling was performed in 30 cases of advanced BTCs, with a subsequent indication to targeted therapy in 60% of the patients. However, only one patient achieved a stable disease, probably due to the long time between surgery and molecular profiling and molecular changes that may have occurred in the meantime [70]. Nevertheless, Lowery and colleagues [71] reported the evidence of a response or clinical benefit in 64% of patients treated with matched targeted therapy after the identification of targetable genetic alterations by next-generation sequencing (NGS). In this study, NGS appears as a useful tool to allocate patients to a specific treatment in a clinically appropriate timeframe, but validation in wider cohorts is needed. The feasibility and clinical utility of prospective molecular profiling is currently under investigation (NCT04318834), and a multicenter, randomized, controlled phase II clinical trial (NCT02836847) is evaluating the response to GEMOX combined with targeted therapies in p/dCCA and GBC patients, after defining their genomic, proteomic and IHC profiles. These kinds of studies, together with the results of ongoing phase III clinical trials, would probably lead to filling the evidence gap in favor of molecular analyses and effective targeted therapies in CCA. In fact, the high intertumoral heterogeneity, largely demonstrated based on single-cell studies, suggests that multiple targets deserve to be hinted at contemporaneously in a same subject. Therefore, an effective step towards precision medicine in CCA would be unraveling the underlying molecular mechanisms implied in cholangiocarcinogenesis and in the acquisition of an aggressive phenotype based on clinical histomorphology and multi-omics (e.g., genome, proteome, transcriptome and radiome) characterization/clusterization. It also seems necessary to develop experimental models that accurately reproduce patients’ conditions in vitro and in vivo and thoroughly characterize them by high-throughput technologies, in order to choose the most suitable model for a specific purpose. Reaching a high translational value in the experimental models might be the key to decreasing the discrepancy between preclinical and clinical studies, leading to a higher clinical efficacy of targeted therapy and, ultimately, to a significant improvement in patients’ prognosis.

## 4. Conclusions

The CCA molecular mechanism has aroused growing interest over the years, and it has been studied on several cohorts through different high-throughput techniques and clustering methods. Multi-omics datasets have provided a large amount of information about CCA pathobiology, although not fully elucidated yet. Nonetheless, this approach is challenging because of the wide dimension and high heterogeneity of datasets, the limited number of patients due to CCA generally low incidence and different data-merging methods [47]. Thus, heterogeneity is the key concept that guided our review with relation to the molecular profiling studies we evaluated. While some authors have underlined anatomical-based signatures, others have shifted the focus of their attention to an etiology-based approach, displaying how risk factors may drive certain molecular patterns. However, to date, studies that specifically analyze the impact of emerging risk factors, i.e., DM2 and NAFLD, on CCA molecular features are lacking. Due to the rarity of the disease, it is difficult to enroll a significant number of patients in clinical trials and translationalstudies to analyze the role of each pathogenetic mechanism. For this purpose, a multi-institutional effort is required not only to widen the studies cohorts but, also, to reach a consensus on morpho-molecular classification of the CCA samples and experimental models. Evidently, a close cooperation between basic scientists, pathologists and clinicians is fundamental to aim at an integrated translational approach, which appears necessary for precision medicine.

The de facto poor translatability of preclinical studies and lack of patient stratification in clinical trials have contributed to the disappointing results of several therapeutic strategies so far tested in CCA. While, in various clinical trials, patients have been treated with targeted therapies without a previous molecular characterization, the risk of a molecular-centered approach is, conversely, to neglect the tumor pathobiology and clinical background, overestimating the prognostic value of molecular signatures.

Hence, we propose an integrated strategy based on the correlation between the etiological, clinical, histological, radiological and molecular findings. Analyzing the clinical–epidemiological and histological features, correlating them to the underlying molecular mechanisms and reproducing the interactions between them in experimental models may be the keys to develop more efficient diagnostic and therapeutic tools. The identification of molecular alterations associated with specific risk factors/underlining liver diseases, clinical presentation and histological and radiological patterns would allow a targeted molecular analysis, instead of time-consuming and expensive high-throughput profiling method and, ultimately, proper patient allocations in clinical trials.

Acknowledging the complexity and heterogeneity of this disease and bearing it in mind when designing both preclinical and clinical studies represent the first step towards effective precision medicine in CCAs, which seems increasingly probable in the near future.

## Figures and Tables

**Table 1:** Comparison of the main polyetiological studies concerning CCA molecular clustering, considering samples features, high-throughput techniques, molecular signatures, clusters, and potential targeted therapy related to driven genetic alterations.

Study	Nakamura et al., 2015 [23]	Wardell et al., 2018 [24]	Sia et al., 2013 [26]	Nepal et al., 2018 [29]	Montal et al., 2020 [27]	Jusakul et al., 2017 [36]
**Samples features**	145 iCCA, 86 p/dCCA, 29 GBC; surgical specimens; Japanese cohort; fluke-	136 iCCA, 109 pCCA, 101 dCCA, and 66 GBC/CDC; surgical specimens; Japanese and Italian cohort	149 iCCA; surgical specimens; American, Italian and Spanish cohort genes; poorest prognosis	496 iCCA	189 p/dCCA; surgical specimens; American, Spanish and Swiss cohort	489 samples from Brazil, China, France, Italy, Japan, Romania, Singapore, South Korea, Taiwan, Thailand 133 Fluke+, 39 HBV-HCV+, 5 PSC+
**High throughput technique**	Whole-exome and transcriptome sequencing	Whole-exome sequencing (n = 107), whole-genome sequencing (n = 39), and targeted sequencing (n = 266)	Whole-genome gene expression microarray profiles, SNP array, and mutation analyses	WES (277), TES (150), genome-wide DNA methylation profiling (69), RNA sequencing (135)	Whole-genome expression profiling, targeted DNA sequencing	WGS (71), targeted sequencing (188), published exome sequencing (200), SNP arrays (175), DNA methylation arrays (138), gene expression arrays (118)
**Driver genes mutations ^1^**	**TP53** (23% iCCA, 26% p/dCCA) **KRAS** (25% iCCA, 12% p/d CCA), **ARID1A** (15% iCCA, 7% p/dCCA), **SMAD4** (10% iCCA, 10% p/dCCA), **BAP1** (12% iCCA, 3% p/dCCA), **PIK3CA**(8% iCCA, 4% p/dCCA), **ARID2** (4% iCCA, 5% p/dCCA)	**TP53** (26%), **KRAS** (17%), **SMAD4** (8%), **NF1** (6%), **ARID1A** (6%), **PBRM1** (6%), **KMT2D** (6%), **ATR** (6%)		**IDH1** (14%), **TP53** (13%) and **KRAS** (12%)	**KRAS** (36.7%), **TP53** (34.7%), **ARID1A** (14%), **SMAD4** (10.7%). Main altered pathways: **RTK-RAS-PI3K** (53%), **TP53- RB** (47%), **histone modification** (22%) and **TGFβ** (18%)	**TP53** (32%) **ARID1A** (17.4%) **KRAS** (16.5%) **SMAD4** (13%) **BAP1** (8.5%) **APC** (7.1%) **PBRM1** (6.5%) **ELF3** (6.3%) **STK11** (5%)
**Molecular clusters**	**Cluster 1:** mostly p/dCCA, negative enrichment of RAS and MAPK activation signatures, best prognosis **Cluster 3:** mostly iCCA, BAP1, **IDH1** and NRAS mutations and **FGFR2 **fusions **Cluster 4:** enrichment of immune system and antiapoptotic genes; poorest prognosis	**Signature A** (5-methylcytosine deamination), **Signature B** (AID/APOBEC deaminases), **Signature C** (nucleotide excision repair deficiency)	**Proliferation** (62%): enrichment of **EGFR**, RAS, MAPK, **AKT**, MET, **VEGF**, PDGF, and HDAC1 pathways; poorest prognosis **Inflammation** (38%): overexpression of Th2 cytokines (IL4, IL10) and pSTAT3, and downregulation of Th1 cytokines	**IDH group:** BCLAF1, ARID1A, BAP1, enriched for metabolic pathways, including glutathione metabolism and citrate cycle. **KRAS group:** SMAD4; **EGFR**, **VEGF** and **actin cytoskeleton rearrangement** **TP53 group:** PTEN, RB1, LATS2, MAPK, WNT **Udt group:** KDM6B, **mTOR** signaling	**Metabolic** (18.7%): HNF4A = key regulator, HDAC6. **Proliferation** (22.5%): overexpression of CSNK2A1, MYC targets, activation of cell cycle signaling and DNA repair pathways, enrichment of Ras/MAPK and **AKT/mTOR** pathways; **ERBB2 **alterations. **Mesenchymal** (47.3%): TGF-β1 = key regulator; TNFα signaling, and periostin. **Immune** (11.5%): IFN-γ = key regulator; over-expression of **PD-1** and **PD-L1**	**Cluster 1**: enrichment of ARID1A and BRCA1/2 mutations, TP53 mutations and **ERBB2** amplifications **Cluster 2** upregulated CTNNB1, WNT5B and **AKT** expression, downregulation of genes involving EIF translation initiation factors; TP53 mutations and **ERBB2** amplifications **Cluster 3** Upregulation **PD-1**, PD-L2 and BTLA **Cluster 4** BAP1, **IDH1 **mutations, **FGFR** alterations, **PI3K** pathway signatures
**Candidate targeted therapy**	**Ivosidenib** [12] **Enasidinib NCT02273739** **Pemigatinib** [38,39] **Derazatinib NCT03230318 Infigratinib NCT03773302 Futibatinib NCT04093362** **Pembrolizumab NCT04003636 Durvalumab** **NCT03875235 Bintrafusp alfa** **NCT04066491** **mTOR pathway modulators** [37,40]	**Ceralasertib NCT03878095**	**Sorafenib** [20] **Regorafenib** [41] **Cetuximab** [19] **Panitumumab** [42,43] **Erlotinib** [44,45] **mTOR pathway modulators** [46,47] **Bevacizumab** [18]	**Ivosidenib** [12] **Enasidinib NCT02273739** **Cetuximab** [19] **Panitumumab** [42,43] **Erlotinib** [44,45] **Bevacizumab** [18] **Microtubule-targeting drugs** [29] **mTOR pathway modulators** [46,47]	**mTOR pathway modulators** [46,47] **Trastuzumab** **NCT03613168** **Lapatinib** [48] **Varlitinib NCT03093870 ** **Pembrolizumab NCT04003636 Durvalumab** **NCT03875235 Bintrafusp alfa** **NCT04066491** **Nivolumab** **NCT02834013**	**Trastuzumab** **NCT03613168** **Lapatinib** [48] **Varlitinib NCT03093870** **mTOR pathway modulators** [46,47] **Nivolumab** **NCT02834013** **Ivosidenib** [12] **Enasidinib NCT02273739** **Pemigatinib** [38,39] **Derazatinib NCT03230318 Infigratinib NCT03773302** **Futibatinib NCT0409336**

^1^ Most frequent genetic alterations (>5% recurrence) or targeted genes.

## Data Availability

Not applicable.
